# Electrical tunable topological valley photonic crystals for on-chip optical communications in the telecom band

**DOI:** 10.1515/nanoph-2022-0169

**Published:** 2022-08-12

**Authors:** Zhipeng Qi, Guohua Hu, Chunyu Deng, Hao Sun, Yaohui Sun, Ying Li, Bo Liu, Yu Bai, Shuaidong Chen, Yiping Cui

**Affiliations:** School of Physics and Optoelectronic Engineering, Nanjing University of Information Science & Technology, Nanjing 210044, China; Advanced Photonics Center, School of Electronic Science and Engineering, Southeast University, Nanjing 210096, China; School of Physical and Mathematical Sciences, Nanyang Technological University, 50 Nanyang Avenue, 639798 Singapore, Singapore

**Keywords:** light modulation, optical communications, robust optical transport, telecom band, topological photonics, valley kink state

## Abstract

On-chip optical communications are in increasingly demand for low-loss, small-footprint and power-efficient waveguiding solutions in the telecom band. However, most integrated optical circuits suffer from high propagation loss and low integration degree. Through manipulating the valley-dependent topological phase of light, we have experimentally demonstrated both robust optical transport and electrical modulation of lightwaves at telecom wavelengths in the valley photonic crystals. With the adoption of valley kink states, the 25 Gbit/s optical signal at 1550 nm is successfully transmitted through a highly twisted interface. Furthermore, an extreme high data rate of 100 Gbit/s is demonstrated with such topological waveguide by wavelength division multiplexing. The electrical tunability of the topological modulators based on thermo-optic effect is also verified, opening a novel route towards active valley kink photonic devices. Our study shows a great possibility of making use of the topological protection in building up high-speed datalinks on a chip.

## 1 Introduction

Valley, termed by local energy extrema of the Bloch band, is a novel degree of freedom (DoF) that offers a reliable way for the manipulation of light [1, 2]. Without the demand of strong spin–orbit interactions, the interband energy flow between different valleys could support information processing under the protection of band topology [3]. Generally, the valley-dependent topological phase transitions for the Bloch waves could be achieved by breaking the spatial inversion (or mirror) symmetry of the periodic structures composed of two-dimensional (2D) honeycomb lattices [4, 5]. At the domain walls or interfaces between two bulk regions with opposite valley-Chern numbers [6], topologically-protected valley kink states emerge [7, 8], which are robust against defects, impurities and sharp bends owning to their bulk-edge correspondences [9]. With this concept, valley photonic crystals (VPCs) characterized by nonzero Berry curvatures at the *K* and *K*′ points were proposed to control the spin and topology of lightwaves [10], showing excellent performances in various nanophotonic devices, such as lasers [11–14], splitters [15–19], and delay lines [20–22].

The application of valley DoF in photonics integration has revolutionized traditional integrated optical devices, enabling disorder-immune and backscattering-free [23–25]. This property prevents optical signals from high losses and severer distortions [26–32], allowing for the miniaturization of optical devices and the monolithic integration of numerous photonic components. Both tortuously-shaped optical paths [33, 34] and heterogeneous channel intersections [35, 36] have been achieved with topologically-protected valley kink states, which is highly desirable for the routing, resonating and refracting of photons on an silicon-on-insulator (SOI)-based photonic integrated circuits (PICs). In addition to robustness, valley kink states with single-mode and linear-dispersion properties are promising for large-bandwidth optical communications [37]. Recently, on-chip terahertz band communications with an extreme high data rate close to 108 Gbit/s [38] were demonstrated in all-Si VPCs, showing great potentials for the uses in the next-generation (6G) mobile communication networks. Moreover, the latest studies show that VPCs could support robust optical transports at near-infrared regimes [39–43], paving the way for the realization of topologically-protected telecom band communications on the SOI platform. Meanwhile, active VPCs have attained more and more attentions due to their on-chip signal processing capabilities [44] in addition to passive ones. Although the mechanically [45–48] and optically [49–51] tunable topological devices have been reported, it still remains challenging to achieve the electrical control of the valley kink states in the SOI-based VPCs.

Here, we design and fabricate the SOI-based VPCs to experimentally demonstrate the transport and modulation of optical signals in the telecom band. In the experiments, we exploit the topologically-protected valley kink state with single-mode and linear-dispersion properties to realize robust optical transport at telecom wavelengths. Similar to the straight Si waveguide, our VPCs could support high-speed data transfers with low bit error rates (BERs), which are also promising in on-chip wavelength division multiplexing (WDM) systems. Additionally, the electrical tunability of the VPC device is enabled by the thermo-optic (TO) effect of the Si, which is initiated by the heat electrodes fabricated on the upper cladding layer. The heat-induced refractive index (*RI*) change results in the shifts of the valley kink state, which in turn affect the phase of the resonant valley kink mode. As the proof of this concept, we modulate the transmitted power in the micro-ring modulator (MRM) based on the manipulation of topological resonating of photons in the triangular-shaped cavity. The proposed VPCs are totally compatible with the complementary metal-oxide semiconductor (CMOS) fabrication process, making a critical step to the practical applications of topological PICs in optical communications and quantum information.

## 2 Results and discussion

### 2.1 Design of SOI-based VPCs

**Figure 1:**
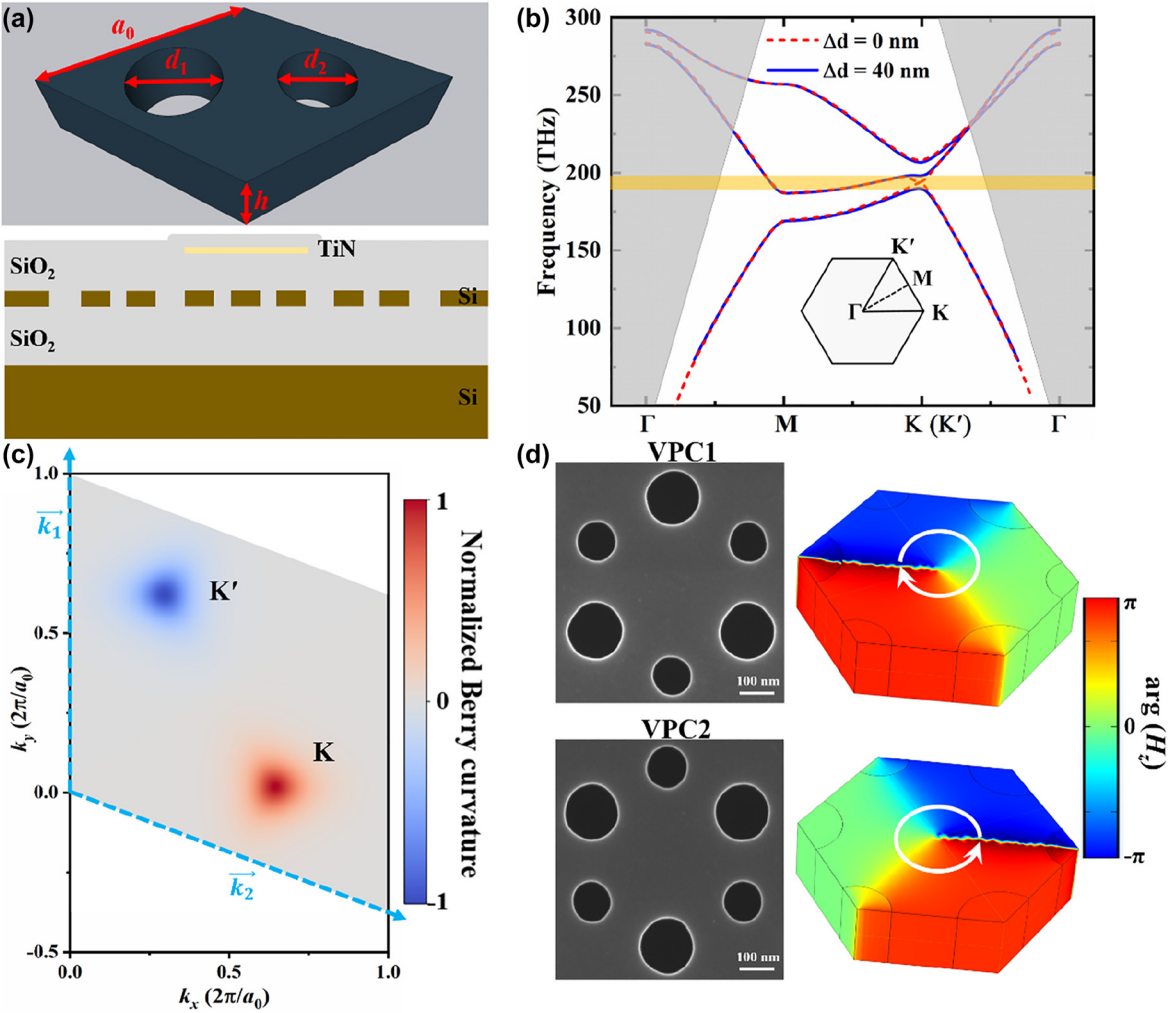
The configuration and band diagram of the proposed VPC. (a) Three-dimensional (3D) schematic view of the rhombic unit cell of lattice (upper), comprised by two SiO_2_ holes with different diameters. The cross-sectional view of the VPC with a heat electrode (lower). (b) Bulk band diagrams for the unperturbed (red dashed line) and perturbed (blue solid line) VPCs. The inset shows the FBZ of the proposed VPC. The grey region represents the radiation zone. (c) Normalized valley-dependent Berry curvatures of the lowest band near the *K* and *K*′ points in the momentum space. And ${\vec{k}}_{1}$ and ${\vec{k}}_{2}$ are the reciprocal lattice vectors. (d) SEM pictures and *H*_*z*_ phase vortex profiles at the *K* valley of the unit cells comprised by two inequivalent SiO_2_ holes for VPC_1_ and VPC_2_, respectively.

The VPCs are fabricated on an SOI substrate with a 220 nm-thick top Si layer and a 2 um-thick buried oxide (SiO_2_) layer. As schematically shown in Figure 1(a), the proposed VPC is composed of the MoS_2_-like 2D honeycomb nanostructure with a lattice constant *a*_0_ = 408 nm, whose unit cell contains two SiO_2_ holes with the same thickness of *h* = 220 nm and different diameters of *d*_1_ = 150 nm (hole A) and *d*_2_ = 110 nm (hole B). And a 2 um-thick SiO_2_ layer is deposited onto the VPC as the upper cladding layer. Here, we only consider the transverse electric (TE)-like optical waves which propagate in the *x*-*y* plane and are highly confined in the *z* direction. When the spatial inversion symmetry is preserved with *d*_1_ = *d*_2_ = 130 nm, the VPC is a graphene-like photonic crystal with *C*_6_ symmetry, displaying a pair of Dirac points at 194 THz in the momentum space. Once the spatial inversion symmetry is broken with the introduction of structural perturbations (*i.e.*, *d*_1_ ≠ *d*_2_), the lattice symmetry of the VPC reduces to *C*_3_, leading to the lift of degeneracy and the bandgap opening (190–198 THz) at the *K* and *K*′ points, as illustrated in Figure 1(b). Even though the fabrication errors of ±10 nm are taken into account, the mirror-symmetry breaking is still realized for such hexagonal lattice, guaranteeing the formations of *K* and *K*′ valleys.

The topological properties of the proposed VPC are correlated with the Berry curvature (Ω) in the first Brillouin zone (FBZ). Based on the finite element method (FEM), the distribution of the local Berry curvature for the lowest band can be numerically calculated. From Figure 1(c), we could observe nonzero Berry curvatures with opposite signs localized around the *K* and *K*′ points, respectively. Although the global integration of Berry curvature over the entire FBZ is zero because of the protection of time-reversal symmetry, the valley Chern number is nonzero, which is given by the integral carried out in half of the Brillouin zone (HBZ) around the *K* and *K*′ points [52], *i.e.*, $C_{K/K^{\prime }}=\frac{1}{2\pi }\int_{\text{HBZ}}\Omega \left(\overset{⇀}{k}\right)dk_{x}dk_{y}=\frac{1}{2}\mathrm{sgn}\left(\Omega_{K/K^{\prime }}\right)$ . Here, *C*_*K*_ = 1/2 and *C*_*K*′_ = −1/2, retrieving the topologically nontrivial phase [53]. Figure 1(d) shows the structural details of VPC_1_ and VPC_2_. They are the mirror-symmetry partners of each other. The phase profiles of the *z*-oriented magnetic field (*H*_*z*_) for the lowest bands of the VPC_1_ and VPC_2_ at the *K* valley are shown in Figure 1(d). As a result of the uniformity of the TE waves, there is no significant change in the *H*_*z*_ phase along the *z* direction. For the VPC_1_, the *H*_*z*_ phase profile exhibits a clockwise circular phase vortex around the center of the unit cell, associated with the right-hand circular polarization (RCP). On the contrary, the *H*_*z*_ phase increases counterclockwise by 2*π* around the center of the unit cell for the VPC_2_, which can be attributed to the left-hand circular polarization (LCP). Despite VPC_1_ and VPC_2_ have the same band structures, the distributions of their Berry curvatures are 180°-rotated because the *K* and *K*′ points are swapped under the spatial inversion. The difference in the valley Chern numbers across the interface between VPC_1_ and VPC_2_ can be obtained by $\left|\Delta C^{K/K\prime }\right|$ = $\left|C_{\text{VPC}1}^{\;K/K\prime }\right.$ – $\left.C_{\text{VPC}2}^{\;K/K\prime }\right|$ = 1, indicating the topological phase transition [54]. Therefore, topologically nontrivial valley kink states could exist in the bandgap at the interface between two bulk regions that are constructed by the VPC_1_ and VPC_2_, respectively.

### 2.2 Valley kink states for on-chip optical communications

**Figure 2:**
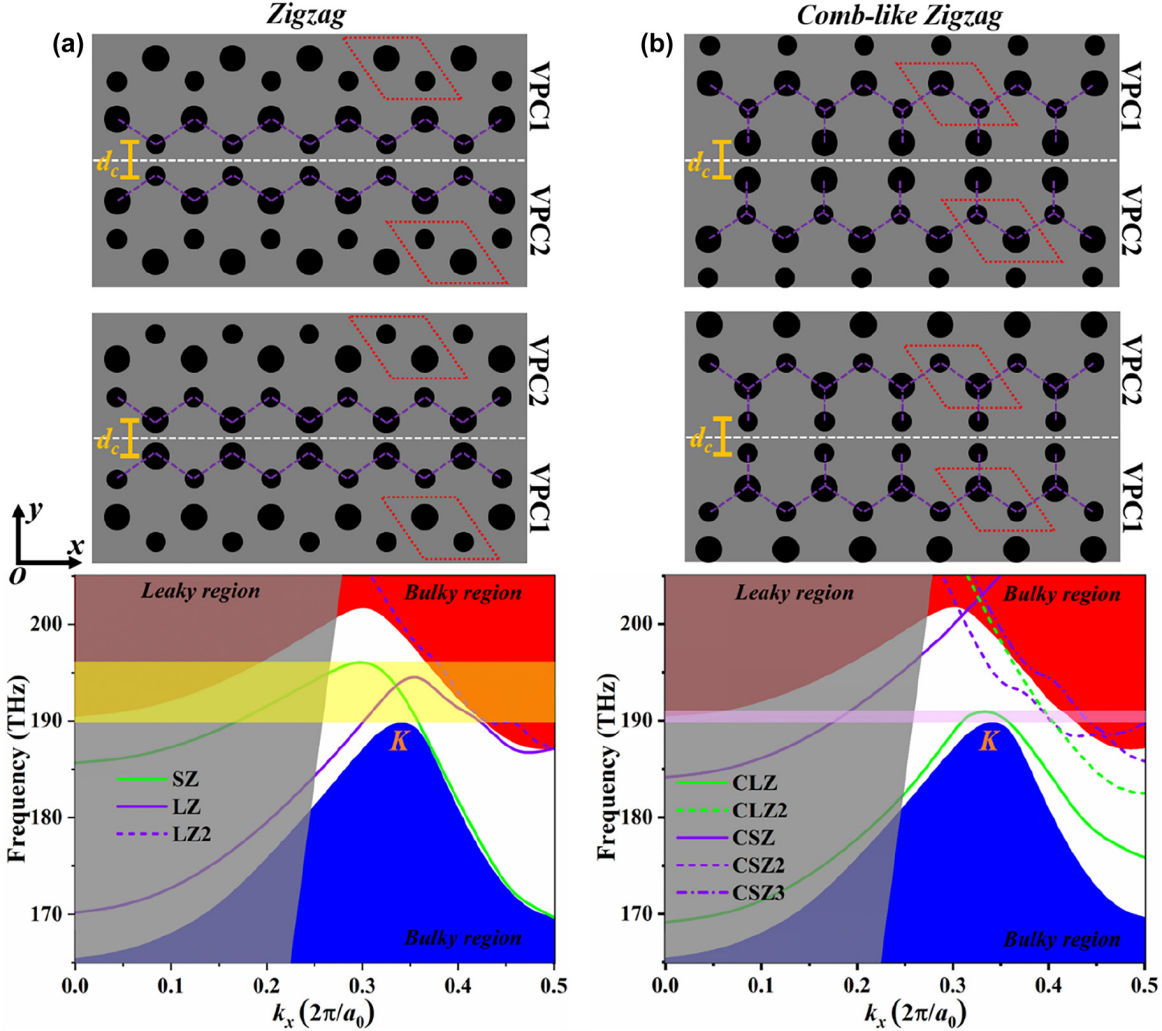
Schematics of valley-dependent interfaces and dispersions of valley kink states for (a) the ‘zigzag’ and (b) the ‘comb-like zigzag’ domains walls with *d*_*c*_ = 235 nm. Red region: TE-like valence band. Blue region: TE-like conduction band. Grey region: light cone of leaky modes. Yellow region: the overlap between the valley kink state for the SZ-interface and the bandgap at the *K*/*K′* point. Magenta region: the overlap between the valley kink state for the CLZ-interface and the bandgap at the *K*/*K′* point.

According to the above analysis, we combine VPC_1_ and VPC_2_ to construct domain walls supporting the propagation of lightwaves, as depicted in Figure 2(a) and (b). There are two fundamental types of combined patterns: the small zigzag (SZ)-interface where the small holes in the zigzag edges of VPC_1_ and VPC_2_ are close to each other and the large zigzag (LZ)-interface where the large holes in the zigzag edges of VPC_1_ and VPC_2_ are close to each other. In addition, there are also two kinds of derived structures based on the SZ- and LZ-interfaces: the combo-like small zigzag (CSZ)-interface where the small holes connected to the LZ edges of VPC_1_ and VPC_2_ are close to each other and the combo-like large zigzag (CLZ)-interface where the large holes connected to the SZ edges of VPC_1_ and VPC_2_ are close to each other. Moreover, the distance (*d*_*c*_) between the adjacent large or small holes along the interface is set to be 235 nm. In our study, the domain wall is assumed to be periodic along the *x* direction and centered along *y* = 0.

Our simulation results show that there are topologically-protected valley kink states locked to the *K* and *K*′ valleys emerging at these interfaces within the bandgap region of 190–198 THz, which are consistent with the bulk-boundary correspondence. A certain wavevector only correspond to a single topologically nontrivial valley kink state (solid lines). Besides, we can also observe the topologically trivial photonic states (dashed lines) for the LZ-, CSZ-, and CLZ-interfaces. They are similar to the ‘defect’ photonic states, which are not under the protection of band topology. The propagation of TE modes with the total internal refraction in the direction normal to the Si slab is not allowed in the leaky region (*i.e.*, above the light line). In the frequency region below the bandgap (<190 THz), the TE mode leakage is enhanced due to the light scattering and the inevitable coupling to the bulk modes, resulting in the increase of total loss. To meet the requirements of on-chip optical communications, the single-mode and linear-dispersion properties of the photonic states are highly required for the purpose of eliminating the optical delay at different frequencies and avoiding the multi-mode competition during the data transfer [23]. In addition, the topologically-protected valley kink states are robust against any structural defects or disorders [39], which could help to improve the fabrication tolerances. For the SZ-interface, the corresponding band diagram verifies the existence of a single quasilinear dispersed (190–196 THz) valley kink state within the bandgap. For comparison, there is a highly bent dispersion curve of the valley kink state for the CLZ-interface ranging from 190 THz to 191 THz, indicating an extreme large group index (*n*_*g*_) for the CLZ valley kink mode. This not only produces localized optical waves but induces a large modal mismatch between the Si waveguide and the CLZ-interface, resulting in low optical transmissions.

**Figure 3:**
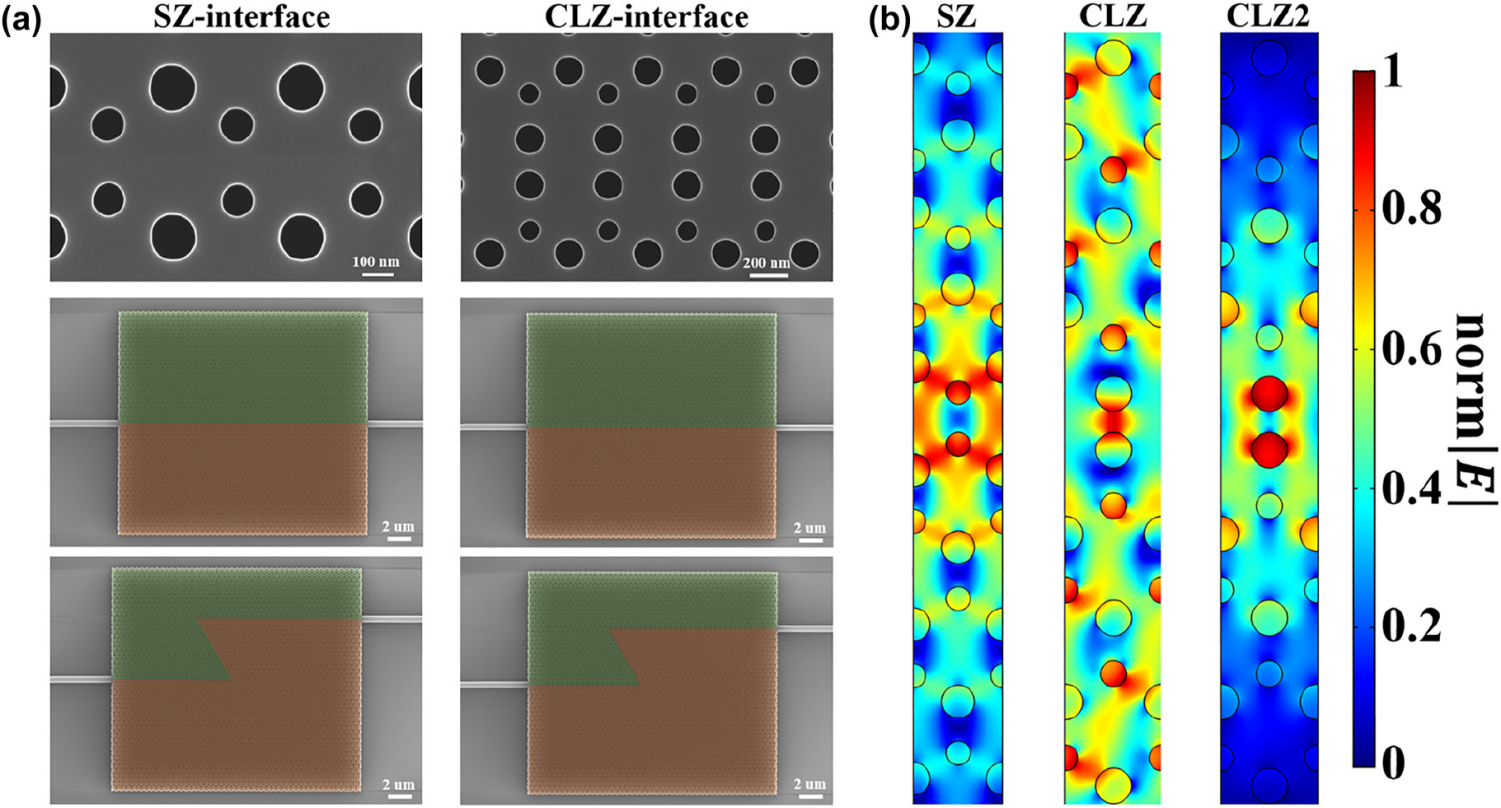
Analysis of optical modes in the topological interfaces. (a) SEM images of the straight and the Z-type domain walls constructed by the SZ- and CLZ-interfaces. (b) Normalized electric field (norm│*E*│) distributions for the simulated optical modes supported by the SZ- and CLZ-interfaces, respectively.

In order to experimentally demonstrate the on-chip robust optical transport in the telecom band, we manufacture the straight and Z-type domain walls in the VPCs based on the SZ- and CLZ-interfaces, as illustrated in Figure 3(a). These structures are fabricated with electron-beam lithography (EBL) followed by dry plasma etching onto the Si layer. The TE-polarized continuous waves (c.w.) at telecom wavelengths (1500–1600 nm) are coupled to the 450 nm-wide input Si rectangular waveguide by utilizing the on-chip Si grating, the polarization controller (PC) and the single-mode optical fiber, and then launched into the VPC part from the left end of the interface. After propagating through the VPC part, the lightwave is coupled into the output Si waveguide at the right end of the interface and collected by another optical fiber, which can finally be detected by the photodetector (PD). From Figure 3(b), we can see the distributions of the normalized electric field (norm $\left|E\right|$ ) of the optical modes supported by the SZ- and CLZ-interfaces, respectively. For the SZ-interface, there is only one valley kink mode confined around the domain wall symmetrically, of which electric field penetrate deeply into the bulk parts. According to the topological valley-locked waveguides [55], CLZ-interface can be regarded as a specific kind of SZ-interface characterized by two large SiO_2_ holes added inside the SZ-interface with a larger width. The domain wall formed by the CLZ-interface could support two kinds of optical modes (CLZ and CLZ2 modes). The CLZ mode is the topologically-protected valley kink mode, whose distribution is analogous to that of the SZ mode. Unlike the topologically nontrivial mode (*i.e.*, CLZ mode), the trivial mode (*i.e.*, CLZ2 mode) is tightly bounded by the ‘defects’ (*i.e.*, large holes) along the interface with only a few modal energies penetrating into the surrounding bulk parts, thus displaying nontrivial topological properties.

**Figure 4:**
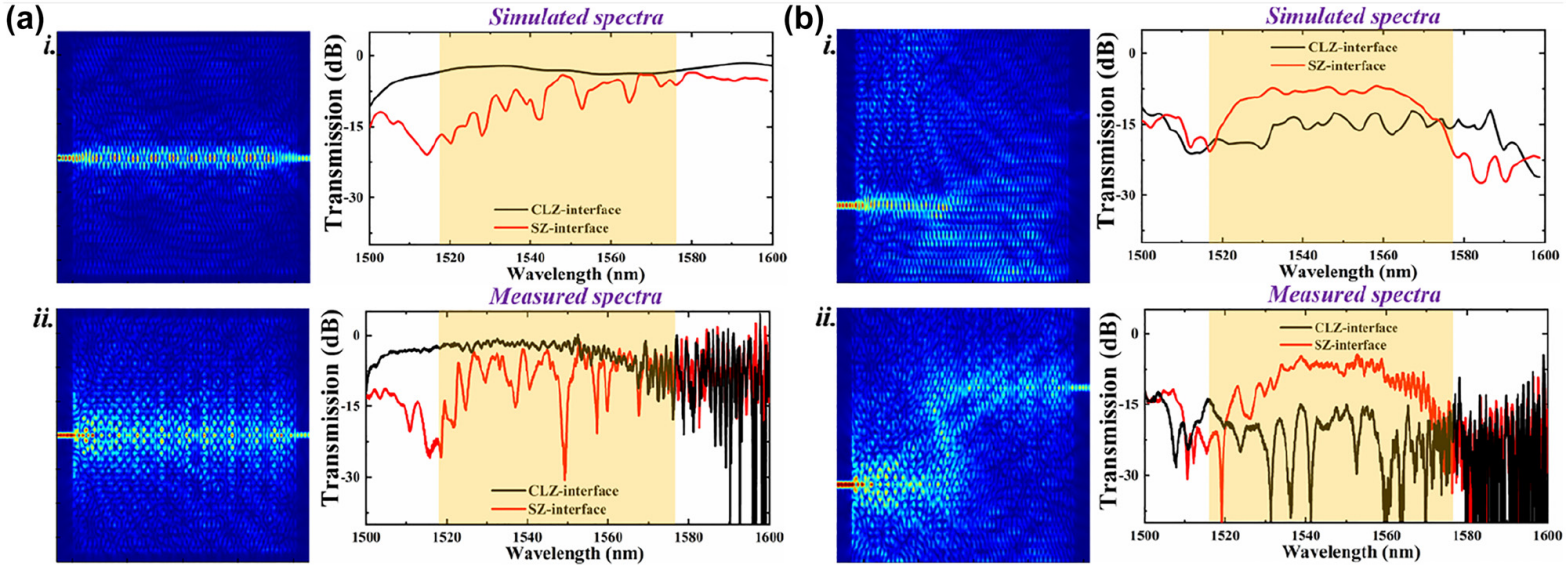
Experimental demonstrations of the optical transports along the domain walls formed by the SZ- and CLZ-interfaces. (a) The simulated electric field distributions in the straight domain walls constructed by the (i) CLZ- and (ii) SZ-interfaces at 1550 nm (left). Simulated as well as measured transmission curves for the straight domain walls formed by the (i) CLZ- and (ii) SZ-interfaces (right). (b) The simulated electric field distributions in the Z-type domain walls constructed by the (i) CLZ- and (ii) SZ-interfaces at 1550 nm (left). Simulated as well as measured transmission curves for the Z-type domain walls formed by the (i) CLZ- and (ii) SZ-interfaces (right). Yellow region: photonic bandgap at the *K*/*K*′ point.

Figure 4(a) and (b) show the simulated as well as measured transmission spectra for the straight and Z-type domain walls constructed by the SZ- and CLZ-interfaces in the wavelength range from 1500 to 1600 nm. It should be noted that all the transmission spectra are normalized to the results measured from the straight Si waveguide located near the test samples. As we can see, the experimental transmission spectra are in good agreement with the simulated ones. Within the bandgap (light yellow region), the transmission curve is kept with a high and flat level for the straight domain wall constructed by the CLZ-interface. However, there is a low optical transmission in the wavelength range of 1515–1525 nm for the straight domain wall constructed by the SZ-interface because neither topologically non-trivial nor trivial optical modes can be excited. Additionally, the transmission maintains high in the spectrum between 1530 and 1575 nm in the Z-type domain wall formed by the SZ-interface, while it drops dramatically with the wavelength exceeding 1575 nm because of the mode coupling into the bulk parts around the sharp corners. The robust optical transports could only be realized in the bandgap region, where the coupling between the *K*/*K*′ valley and valley kink state is eliminated. For the straight SZ-interface, there are no defects or sharp corners influencing the propagating waves, thus showing a relatively high optical transmission even beyond 1575 nm. Besides, one can observe obvious Fabry–Perot (F-P) resonant dips in the spectrum for the straight domain wall formed by the SZ-interface, originating from the incomplete evolution of TE mode to valley kink mode. The incomplete evolved optical modes are topologically trivial without the unidirectional property, which can be easily reflected by the boundaries between the VPC part and Si waveguides. On the contrary, there are much fewer and weaker resonant dips in the spectrum for the Z-type domain wall formed by the SZ-interface within the linear dispersion region of the valley kink state (190–194 THz). This is because only topological valley kink modes can propagate through this highly twisted waveguide, contributing to few reflections. On the other hand, the valley kink state for the CLZ-interface is nonlinearly dispersed from 1570 to 1578 nm, accompanied with large *n*_*g*_. The mismatch between the TE mode and the valley kink mode in the CLZ-interface contributes to a significant light reflection and refraction at the boundaries between the VPC and Si waveguides. Outside the region of 1570–1578 nm, only topologically trivial or bulk modes could be excited in the CLZ-interface. Consequently, there are low optical transmissions for the Z-type domain wall constructed by the CLZ-interface both within and beyond the bandgap. In our study, the finite-domain time-difference (FDTD) simulations are performed for all the cases. We can clearly see the distributions of *H*_*z*_ in the straight and Z-type domain walls consisting of both SZ- and CLZ-interfaces. It is evident that the valley kink mode excited in the SZ-interface could propagate through a 45° sharp corner smoothly with almost no reflection and refraction, verifying the possibility of achieving the topologically-protected valley kink states at telecom wavelengths in our topological PICs. However, the measured transmission spectra show the noisy behavior in comparison with the simulated results, which can be attributed to the dark current of the PD and the resonances induced by the Si grating. A small footprint of around 20 × 18 μm^2^ can be guaranteed by such SOI-compatible VPCs, allowing for the integration with various photonic components on a chip.

**Figure 5:**
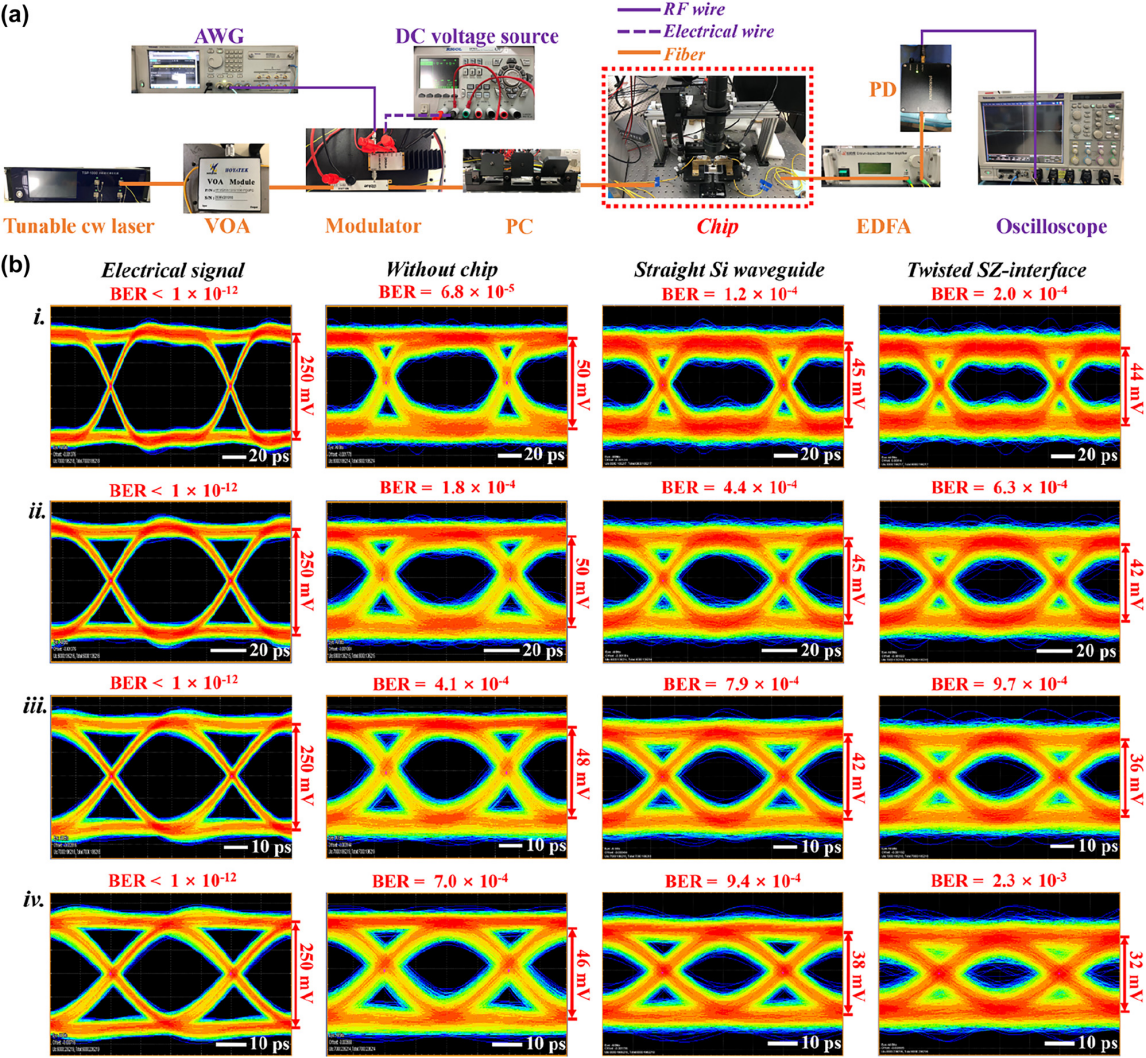
On-chip optical communications. (a) The experimental setup for the optical communication test at 1550 nm. (b) Eye diagrams for the electrical signals generated by AWG (1st column), the data transfers without chip (2nd column), the data transfers in the Z-type domain wall formed by the SZ-interface (3rd column) and the data transfers in the straight Si waveguide (4th column) at data rates of (i) 10, (ii) 15, (iii) 20, and (iv) 25 Gbit/s.

Valley kink states have exhibited remarkable performances in large-capacity and high-quality information transfer at THz band, which are highly suitable for the application in the future 6G networks [37]. To further verify such property in the telecom band, we perform the similar test in our VPC with a highly twisted interface. The experimental setup based on the transmission of the topologically-protected valley kink mode at 1550 nm along the Z-type domain wall constructed by the SZ-interface is shown in Figure 5(a). The none-return-to-zero (NRZ) on-off-keying (OOK) electrical signals are generated by an arbitrary waveform generator (AWG, Tekronix AWG70002A), which are then converted to the optical ones using an external high-speed intensity modulator (iXblue DR-AN-40-MO). To ensure the minimal coupling loss between the fiber and the Si grating, the modulated lightwave at 1550 nm passes through a PC. Finally, the amplified optical signals are firstly collected by the PD and further measured by a real-time oscilloscope (Tekronix MSO73304DX) with the digital signal processing bandwidth up to 40 GHz. A comprehensive measurement is conducted with different bit transfer rates for the proposed VPC device. In addition to the straight Si waveguide and the Z-type domain wall constructed by the SZ-interface, the measured results are also compared in the fields of BERs and eye diagrams between the electrical signal and the data transfer without chip, as depicted in Figure 5(b). When the chip is not inserted into the system, we utilize a variable optical attenuator (VOA) to produce 7 dB loss, which could take place of the insertion loss (IL) for the straight Si waveguide. It can be clearly seen that opened eye diagrams as well as small BERs are achieved at data rates of 10, 15, 20 and 25 Gbit/s for both straight Si and twisted topological waveguides, verifying the possibility of achieving low-error and high-speed data transfer in SOI-based topological photonic circuits. Besides, the eye diagrams and BERs of the transmitted signals for the straight Si and twisted topological waveguides are almost in the same level with those measured from the experimental setup without chip, illustrating that the amounts of the jitter and noise due to on-chip mode losses and distortions are very small. However, the data transfer in the twisted topological waveguide is slightly worse than that in the straight Si waveguide at 10, 15, 20, and 25 Gbit/s, which can be attributed to the coupling loss between the TE mode and the valley kink mode. As a result, it is of great importance to make further improvements on the modal mismatch between the SZ-interface and the conventional Si waveguide. The above experimental results reveal that the topologically-protected valley kink state could act as a reliable information carrier in the telecom band.

**Figure 6:**
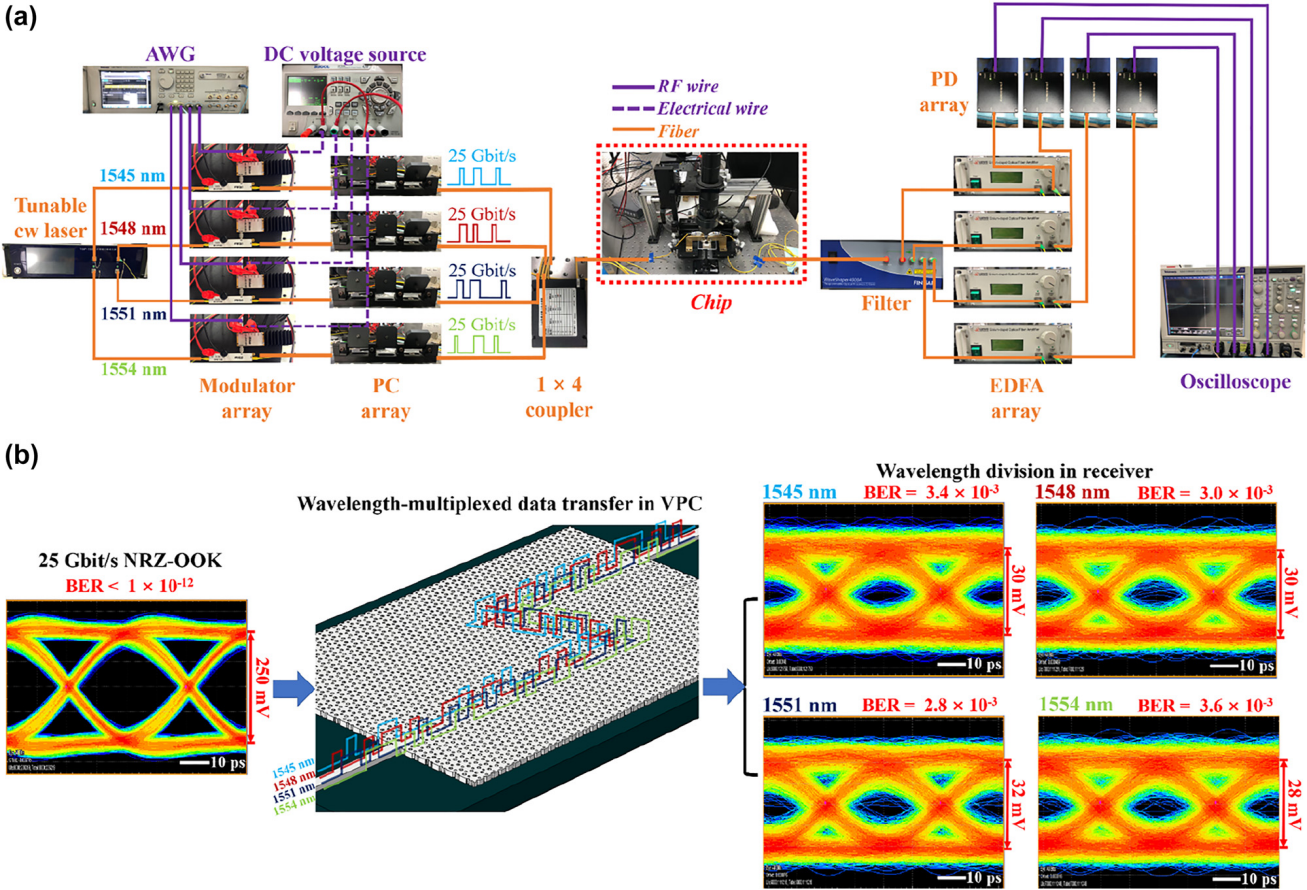
On-chip optical communication in the telecom band based on WDM. (a) The experimental setup for the WDM-based on-chip optical communication test. (b) Eye diagrams and BERs of the 25 Gbit/s arbitrary NRZ-OOK electrical signal (left) and the received data with different wavelengths (right). Schematic diagram of the wavelength-multiplexed data transfer in the Z-type domain wall formed by the SZ-interface (middle).

In Figure 4(b), it is observed that the twisted topological waveguide constructed by the SZ-interface offers a flat and high optical transmission window of 1540–1560 nm, showing a great potential in on-chip WDM data transfer. As shown in Figure 6(a), we have further performed the telecommunication experiment with such topological waveguide based on the WDM setup, which could help to improve the data rate greatly. The arbitrary NRZ-OOK signals at the rate of 25 Gbit/s are loaded onto four lightwaves with the wavelengths of 1545, 1548, 1551, and 1554 nm, respectively. These four optical signals with different wavelengths are multiplexed into one fiber and then coupled into the chip. After propagating through the Z-type domain wall constructed by the SZ-interface, the mixed optical signals are collected by the output fiber and divided by the filter based on wavelengths before reception. From Figure 6(b), one can see opened eye diagrams for the received signals corresponded to 1545, 1548, 1551 and 1554 nm, of which averaged BER is nearly 3.2 × 10^−3^. Therefore, the low-error WDM-based on-chip data transfer with a total rate up to 100 Gbit/s can be achieved with our topological waveguide. It is also possible to obtain the data rate larger than 100 Gbit/s by exploiting more wavelengths (>4) in the telecom band for the on-chip data transfer.

### 2.3 Electrical modulation for the topological MRM

**Figure 7:**
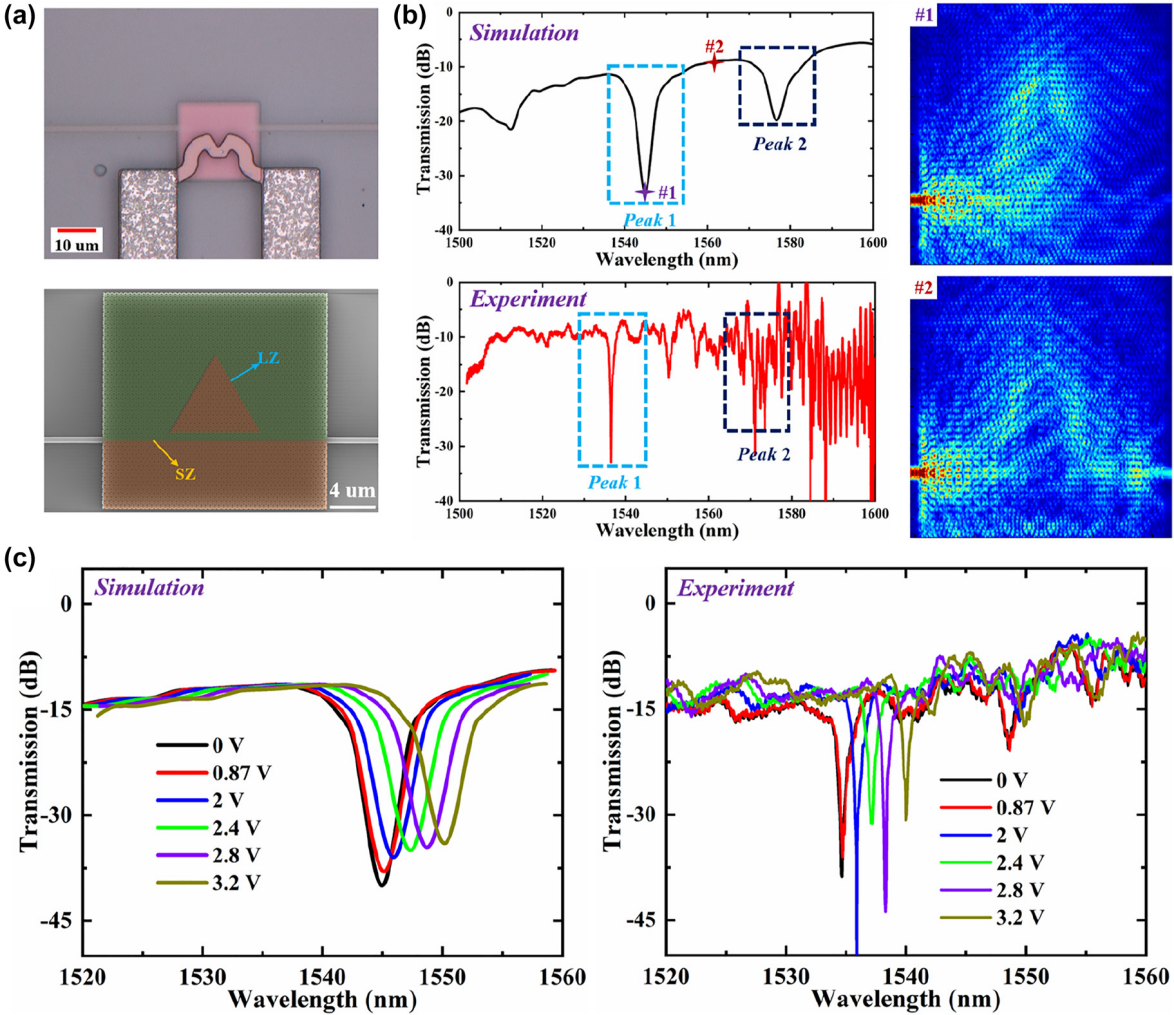
Electrical tuning of the topological MRM. (a) The microscope and SEM images of the VPC-based topological MRM consisting of a straight domain wall constructed by the SZ-interface and a triangular-shaped cavity constructed by the LZ-interface. (b) Simulated and experimental transmission spectra of the MRM in the wavelength range of 1500–1600 nm. The simulated electric field distributions in the topological MRM for the wavelengths corresponded to #1 and #2 shown in the simulated transmission spectrum. (c) Simulated and experimental transmission spectra of the MRM with different applied DC voltages in the wavelength range of 1520–1560 nm.

We have also investigated the electrical tunability of the proposed VPCs fabricated on the SOI substrate, as schematically shown in Figure 7(a). The triangular-shaped cavity with a side length of nearly 8.3 um (indicated in red) is composed of the LZ-interface, which is directional coupled with a straight domain wall constructed by the SZ-interface. Based on the above analysis, there are topologically-protected valley kink modes can be excited in both SZ- and LZ-interfaces, enabling the robust optical transport against sharp corners. As a result, the whispering gallery modes (WGMs) can also be formed in such triangle-shaped cavity. The RI of Si can be controlled by the heat generated by the electrode. Once the RI of Si is changed, the dispersion of the valley kink state for the LZ-interface varies, leading to the shift of the resonant wavelengths. As we can see in Figure 1(a), the heat electrode is made by TiN that possesses good electrical and thermal conductivities and buried into the SiO_2_ upper cladding layer in case of heat dissipation and metal oxidation.

We measure the transmission spectra for this topological MRM by making use of a tunable laser (Santec, TSL-710) with 0.05 nm wavelength resolution and an optical power meter (Santec, MPM-210). As illustrated in Figure 7(b), the measured spectrum is in good agreement with the simulated one except for the slight blueshifts of the resonant wavelengths owning to the fabrication errors (±10 nm). And since the optimal operation window of the Si grating coupler is 1500–1570 nm, the noises are enhanced significantly in the spectrum with the wavelength increasing above 1570 nm. The valley kink mode corresponded to the SZ-interface evanescently couples to the triangular-shaped cavity constructed by the LZ-interface, transforming into the topologically-protected cavity mode. We find such cavity mode could be highly confined with few powers coupling into the output Si waveguide at the resonant wavelengths, operating like a conventional micro-ring resonator. The simulated as well as measured transmission spectra with different DC voltages (*V*_DC_) are shown in Figure 7(c). An obvious transmission dip can be observed at the wavelength of 1534.6 nm when *V*_DC_ is set below 0.9 V, corresponding to the resonant valley kink mode excited in the LZ-interface. Because the vertical distance between the heat electrode and the VPC is close to 2 um, a small voltage (*V*_DC_ < 0.9 V) can hardly generate enough heat to influence the Si layer. With *V*_DC_ increasing above 2 V, we can observe the significant redshift of the resonant wavelength (*λ*_res_), which is caused by the shift in the dispersion of the valley kink state for the LZ-interface. The relationship between *λ*_res_ and *V*_DC_ is almost linear when *V*_DC_ is swept from 2 to 3.2 V with a step of 0.4 V. Here, the averaged wavelength tuning rate (*i.e.*, Δ*λ*_res_/Δ*V*_DC_) within the voltage range of 2–3 V is calculated to be 3.3 nm/V. Besides, the quality (Q) factor for this MRM is about 730, varying very few during the electrical tuning. And the extinction ratio (ER) for this transmission dip is over 25 dB, which is highly attractive for the chip-integrated light modulation.

**Figure 8:**
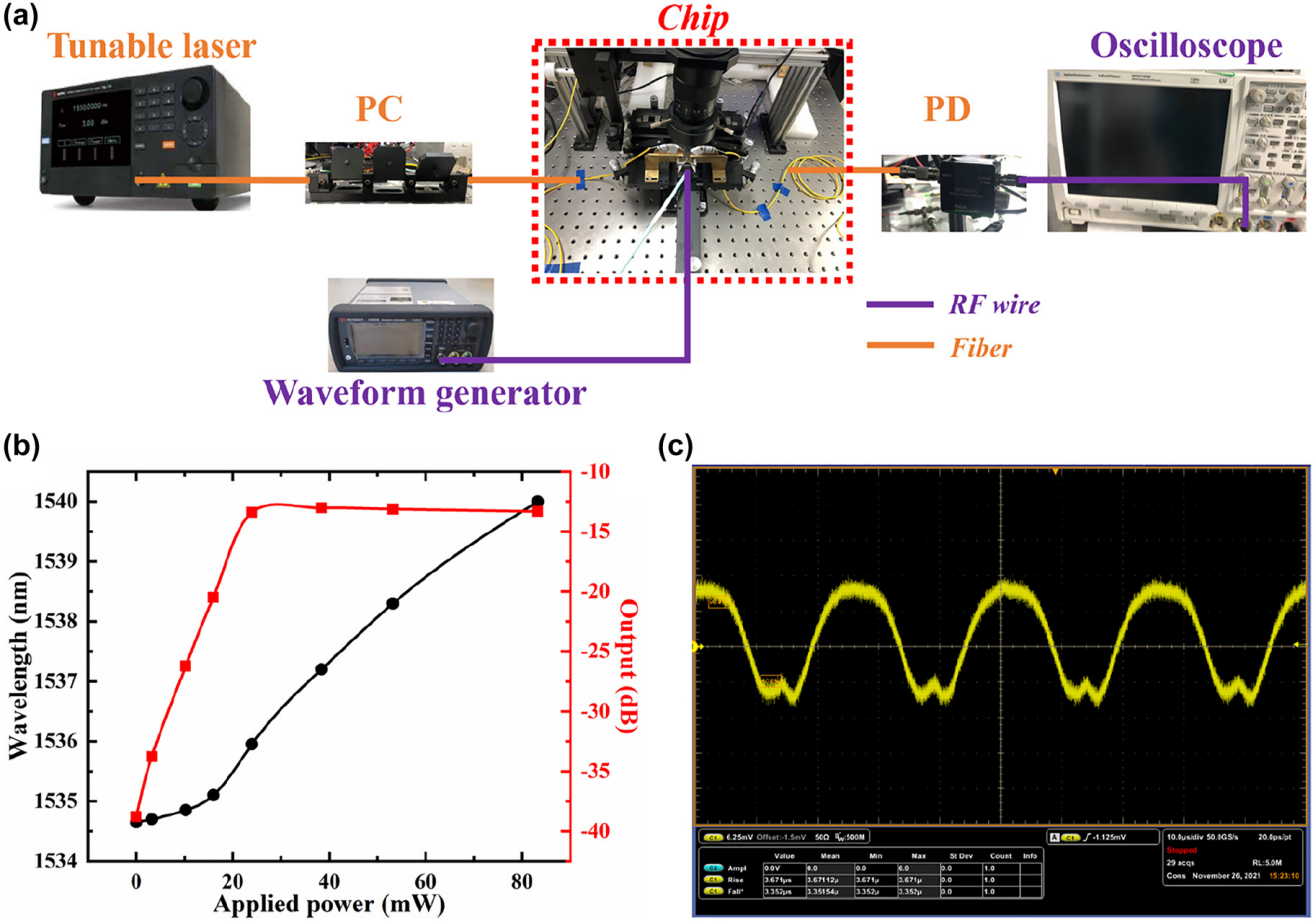
Temporal response of the topological MRM. (a) Experimental setup for the to modulation of the VPC-based topological MRM. (b) The resonant wavelength shift of such MRM and the measured output as a function of the applied electrical power. (c) Dynamic characteristics of the topological MRM on the oscilloscope at a modulation frequency of 40 kHz.

To verify the performance of the real-time on-chip light modulation for such topological MRM, we establish another experimental setup for the signal processing based on the chip-scale electronic-to-optic (EO) conversion, as depicted in Figure 8(a). In Figure 8(b), one can observe the IL of this MRM changes from −40 dB to −13 dB at the input wavelength of 1534.6 nm with the increase of the applied electrical power to nearly 24 mW (corresponding to *V*_DC_ ≈ 2 V). This indicates we could achieve a large modulation depth close to 30 dB with moderate power consumption. In addition, *λ*_res_ shifts from 1534.6 to 1535.9 nm when the applied electrical power is increased from 0 to 24 mW. And it varies slowly in the power range of 0–16 mW with an averaged tuning efficiency of 0.031 nm/mW, which then varies rapidly in the power range of 16–24 mW with a higher averaged tuning efficiency of 0.106 nm/mW. With the consideration of the accuracy limits of ultraviolet (UV) photolithography, there is a transverse offset distance (∼1 um) between the heat electrode and the LZ-interface. This in turn causes even more nonuniform distributions of the heat in the upper cladding and the Si layers, lowering the tuning efficiency. Figure 8(c) illustrates the dynamic characteristic of the proposed topological MRM, revealing that the rise/fall time of the modulated signal can reach 3.5 us. For the conventional SOI-based TO devices, most of them could only obtain the response times of several tens microseconds except by adopting specific configurations such as F-P microcavity (<1 us) [56] and high-efficiency heaters such as NiSi (∼2 us) [57]. Our topological MRM is more compact with a smaller footprint of ∼25 um^2^, ensuring sufficient interactions with the heat field. Nevertheless, the power consumption of this MRM is still relatively high because large amounts of heats are wasted in the cladding layers. Besides optimizing the cladding configurations [58], the power consumption can be decreased to uW scale by enhancing the thermal isolation [59]. The response speed of such MRM could support a modulation rate of ∼140 kbit/s in the telecom band, which is high among the TO devices but still far worse than the electro-optic modulators. Hence, it will be more attractive to apply the proposed MRM in the optical links and routers as an on-chip TO switch. This VPC device exhibits a great possibility for the manipulation of lightwaves protected by the band topology under the TO interaction.

## 3 Conclusions

We hence experimentally demonstrate robust optical transport and TO modulation of topologically nontrivial optical modes in the telecom band by using SOI-based VPCs. In addition to terahertz communications, we show that topologically-protected valley kink states could also support on-chip telecommunications. Through adopting the robustness, single-mode and linear-dispersion properties of the projected valley kink state, the on-chip data transfer with a high rate up to 25 Gbit/s is obtained at the wavelength of 1550 nm, which can be further increased to 100 Gbit/s based on WDM. The proposed topological interfaces show a great potential for the usage in various integrated functional optical components, including splitters, interferometers and optical delay lines. In addition, we also experimentally manipulate the lightwave propagating in the topological MRM based on the TO effect. The rise/fall time of the proposed modulator could reach nearly 3.5 us. Meanwhile, a large ER of more than 25 dB and a moderate power consumption of 24 mW are achieved with our device, paving the way for the applications of SOI-based VPCs in on-chip EO conversions. Our study offers experimental verifications of both on-chip telecommunications and electrical tunability under the protection of band topology, inspiring related investigations of topological valley Hall phase on the SOI platform.
